# Prevalence and characteristics of covert contraceptive use in the Performance Monitoring for Action multi-country study

**DOI:** 10.1016/j.conx.2022.100077

**Published:** 2022-05-06

**Authors:** Dana O. Sarnak, Elizabeth Gummerson, Shannon N. Wood, Funmilola M. OlaOlorun, Simon Peter Sebina Kibira, Linnea A. Zimmerman, Philip Anglewicz

**Affiliations:** a Department of Population, Family and Reproductive Health, Johns Hopkins Bloomberg School of Public Health, Baltimore, MD, United States; b Department of Community Medicine, College of Medicine, University of Ibadan, Ibadan, Oyo State, Nigeria; c Department of Community Health and Behavioral Sciences, School of Public Health, Makerere University, Kampala, Uganda

**Keywords:** Covert contraceptive use, sub-Saharan Africa, Family planning, Partner dynamics

## Abstract

**Objectives:**

A more nuanced understanding of contributors to covert contraceptive use remains critical to protecting covert users and reducing its necessity. This study aimed to examine the overall prevalence of covert use, and sociodemographic characteristics associated with covert vs overt use across multiple geographies in sub-Saharan Africa and Asia.

**Study Design:**

Performance Monitoring for Action (PMA) is one of the few nationally representative surveys that measures covert use across socially diverse contexts via a direct question. Utilizing PMA 2019–2020 phase 1 data from Burkina Faso, Côte D'Ivoire, Kenya, Democratic Republic of Congo (DRC; Kinshasa and Kongo Central regions), Uganda, Nigeria (Kano and Lagos), Niger, and Rajasthan, we estimated overall prevalence of covert use. We conducted bivariate analyses and multivariate logistic regressions for 6 sites, comparing the odds of covert use with overt use among users of contraception by sociodemographic characteristics.

**Results:**

Covert use ranged from 1% in Rajasthan to 16% in Burkina Faso. Marital status was the only sociodemographic characteristic consistently associated with type of use across sites. Specifically, polygynous marriage (compared to monogamous) increased odds of using covertly, ranging from adjusted odds ratio (aOR) of 1.8 [95% confidence interval (CI) 1.2–2.7] in Burkina Faso to 6.2 [95% CI 2.9–13.3] in Kinshasa. Unmarried women with partners or boyfriends were also more likely to be using covertly compared with their monogamously married counterparts in all sites (aORs ranged from 2.2 [95% CI 1.0–4.7] in Uganda to 4.4 [95% CI 1.7–11.0] in Kinshasa).

**Conclusion:**

Understanding factors associated with covert use has programmatic and policy implications for women's reproductive autonomy.

**Implications:**

Covert use is a common phenomenon across most sites, representing a small but programmatically important contingent of users. Family planning providers and programs must protect access to and maintain privacy of reproductive services to this population, but should also focus on creating interventions and environments that support overt use.

## 1 Introduction

Covert use of contraception (i.e., contraceptive use without partner's knowledge) is a longstanding strategy employed by women to achieve the goal of preventing pregnancy, particularly within contexts where systemic gender and power norms enable men to dominate reproductive decision-making, such as in sub-Saharan Africa (SSA) [1], [2], [3], [4]. Given that the Demographic and Health Surveys (DHS) no longer ask women using contraception if partners are aware of their use [1,4] there are few recent studies directly assessing covert use in the SSA context. This gap is of consequence, since understanding the prevalence of and contributors to covert use remains critical to ensuring that providers meet the needs of this unique population.

Previous studies have contextualized women's experiences to explain the circumstances that lead to covert use. A seminal study in Zambia found that women were motivated to use contraception in secret due to concerns about the health and economic welfare of their children; their husbands’ opposition to contraception; pronatalism; and limited or conflictual spousal communication [2]. Another study from Mali showed that covert users were motivated to use discreetly given husband opposition to contraceptive use due to pronatalism, religion, worries about infidelity, and contraceptive-induced side effects [5]. Recent quantitative evidence from Kenya suggests that higher reproductive autonomy is linked with decreased covert use [6]. These 3 studies suggest that covert use is a product of personal concern and gendered disempowerment. By contrast, a recent qualitative study in 4 SSA contexts suggests that covert use may, in fact, be an expression of individual autonomy, as some women perceive contraceptive use as solely their choice [3].

The true prevalence of covert use in a population remains unknown, given the various techniques used to measure it (direct vs indirect), comparison groups (among all users or among all women), and limited evidence base; but as an example, one study estimated covert use among women using modern contraception in 21 sub-Saharan African countries to range from 2% to 69% [1]. Despite the pervasiveness of covert use, few studies have examined differences in user characteristics between covert and overt users. Among those that have been recently conducted, correlates of covert use vary substantially across settings, and include being previously or never married, having low or no schooling or completing higher levels, having multiple sex partners in the past year, not cohabitating with partners, urban residence, working in a nontraditional industry, being less wealthy and experiencing physical abuse [7,8,9].

While these women may be achieving their reproductive goals by avoiding pregnancy, there is evidence that covert use, among those for whom covert use is a product of lack of decision-making power, may be linked to negative impacts on health and well-being. Covert users may be less inclined to seek treatment or switch methods due to side effects than overt users, particularly if side effects pose risk of discovery [2,3,5,10]. Covert use may lead to emotional distress, as it may generate feelings of distrust or fear within a relationship or conflict with the user's faith. [5,9,11,12]. Consequences of discovery may be severe, potentially resulting in physical violence and marital or financial abandonment upon discovery [3,9,13]. With a greater understanding of the profiles of covert users, health care providers can more effectively counsel women, ensure safe continuation of contraceptive methods, and help secure women's privacy.

Currently, the field is lacking comparative, cross-country studies of factors associated with covert use among contraceptive users. Our study is one of few population-level representative studies able to estimate covert use across distinct social and geopolitically diverse contexts. This study aimed to estimate the overall prevalence of covert use and examine sociodemographic characteristics among covert and overt users across multiple nationally representative geographies.

## 2 Materials & methods

### 2.1 Data

We used data from Performance Monitoring for Action (PMA). PMA collects representative data on family planning and contraceptive use in eight geographies in Africa and Asia. PMA uses a multistage stratified cluster design, starting with the random selection of enumeration areas of approximately 200 households based on the relevant national census, followed by the random selection of 35 households within each area. All women ages 15 to 49 living in the selected households and who provide informed consent are interviewed. Datasets are publicly available from the PMA website at www.pmadata.org; detailed information on the study design is provided in Zimmerman et al. [14].

In 2019, PMA changed from a repeated cross-sectional design to a longitudinal household panel. PMA initiated panel data collection (phase 1) in Fall 2019, starting with Burkina Faso (national), Côte d'Ivoire (national), Kenya (national), the DRC (Kinshasa and Kongo Central provinces), Uganda (national), Nigeria (Kano and Lagos regions), Niger (national), and Rajasthan (region). This study uses the data collected at phase 1 only. PMA received ethical approval from institutional review boards in each country including the Comité d'Ethique Institutionnel Pour La Recherche en Santé (Burkina Faso), École Nationale de Statistiques et d'Economie Appliquee of Abidjan (Côte d'Ivoire), Kenyatta National Hospital-University of Nairobi Ethics Research Committee (Kenya), the Comité d'Ethique Ecole de Sante Publique Universite de Kinshasa (DRC), Makerere School of Public Health and the Uganda National Council for Science and Technology (Uganda), Kano State Ministry of Health (Nigeria-Kano); The Lagos State University Teaching Hospital Heath Research Ethics Committee (Nigeria-Lagos), Ministere de la Sante Publique Comite National d'Ethique pour La Recherce en Sante (CNRS) (Niger), Indian Institute of Health Management Research Institutional Review Board for the Protection of Human Subjects (Rajasthan), and the Johns Hopkins Bloomberg School of Public Health (USA).

The present analysis utilized PMA phase 1 data, which had high response rates (<2% refusal in each geography). This analysis is restricted to partnered women who had stayed in the selected household the night before, and who reported currently using contraception at phase 1. Partnered women were defined as currently married or living with a man, or those who were not married or living with a man, but who reported having a current partner. Our final sample sizes are as follows: Burkina Faso (*n* = 1727); Côte D'Ivoire (*n* = 926), Kenya (*n* = 3532); Kongo Central (*n* = 589); Kinshasa (*n* = 890); Uganda (*n* = 1046); Nigeria-Kano (*n* = 125); Nigeria-Lagos (*n* = 533); Niger (*n* = 570); Rajasthan (*n* = 2673).

### 2.2 Measures

Our outcome of interest was type of contraceptive use, a binary variable defined as covert or overt use. We defined covert use via woman's response to the following item: “*Does your partner/husband know that you are using [method]*?,” which was asked of all female-controlled modern (female sterilization; implants; IUD; injectables; pill; emergency contraception; female condom, standard days/cycle beads) or traditional methods (rhythm; other traditional). Women who responded “no” were considered covert users, while women who responded “yes” were defined as overt users. We further classified women as overt users if they were using male-dependent methods (male sterilization, male condoms, or withdrawal), who were not asked the question of whether their partner knows about use. The decision to include users of male-dependent methods and categorize them as overt users follows prior research [2, 7,8].

Our explanatory variables of interest were socio-demographic characteristics that have been shown from previous research to influence contraceptive use, and include urban/rural residence,^1^ age, parity, education, type of partnership (currently married/living with partner and monogamous; currently married/living with partner and polygynous; not married), and wealth tertile [7,8,9]. The wealth tertile variable was calculated at the country level using principal components analysis, and its construction was consistent across each country dataset. Ideally, we would have liked to include more relationship characteristics such as spousal age differentials and relationship duration, as these factors may have bearing on a woman's decision-making power in the relationship as well as childbearing expectations. However, PMA currently only asks how long women who are married/living with their partners have been cohabitating. Therefore, we included this measure in a sensitivity analysis described below.

### 2.3 Analyses

First, we compared socio-demographic characteristics and prevalence of covert use across sites. Next, we compared the characteristics by type of contraceptive use (covert and overt). We used design-based F-statistics to test whether the differences between overt and covert users were significant. Finally, we conducted simple and multiple logistic regressions for each site, comparing the odds of covert use vs overt use (referent), among users of contraception, by socio-demographic characteristics. Only the prevalence of covert use is reported for the Nigerian geographies, Niger, and Rajasthan. RjaaRajasthan Due to the low numbers of covert users (*n* < 75), these geographies were excluded from further bivariate and multivariate analyses.

All analyses were conducted in STATA version 16 [15] and accounted for the multi-stage complex survey design. Analyses are weighted to be nationally representative in all sites except for Kinshasa and Kongo Central, where they are representative of those regions.

## 3 Results

### 3.1 Descriptive statistics—Overall sample

Sample characteristics are presented separately by geography in Table 1. Education levels differ across sites; in Burkina Faso and Niger, over half of respondents report no formal education (55% and 62%, respectively), while in other geographies the percentage is considerably lower, ranging from <1% (Kinshasa) to 43% (Rajasthan). Partnership status varied substantially—the majority of women were married or living with men in monogamous partnerships in all sites, highest in Rajasthan (97%) and Kenya (73%). Proportions of women who reported being in polygynous marriages were highest in Burkina Faso (34%) followed by Uganda (22%). One in 5 or more women in Côte D'Ivoire, Kongo Central, and Kinshasa were not married or living with their partners. Parity also differed across sites. Proportions of nulliparous women were highest in Kinshasa (28%) and Côte D'Ivoire (22%) while those with 5 or more children were highest in Burkina Faso (35%) and Uganda (31%).

**Table 1:** Weighted percentage distributions of characteristics of contraceptive users in Burkina Faso, Côte D'Ivoire, Kenya, Kongo Central, Kinshasa, Uganda, Kano, Lagos, Niger and Rajasthan, 2019/2020

	Burkina Faso	Côte D'Ivoire	Kenya	Kongo Central	Kinshasa	Uganda	Nigeria-Kano	Nigeria-Lagos	Niger	Rajasthan
*n*	2097	1088	3992	721	1060	1247	125	533	570	2673
Characteristic										
*Household characteristics*										
Household wealth										
Lower	29.5	20.3	32.0	23.3	31.9	26.1	9.4	31.6	23.6	30.3
Middle	30.0	34.8	36.1	30.9	33.5	35.0	18.6	31.9	29.7	34.3
Highest	40.5	44.8	31.9	45.8	34.5	38.8	72.0	36.5	46.8	35.4
Residence										
Urban	30.2	63.5	33.2	NA	NA	33.2	65.4	NA	29.8	25.3
Rural	69.8	36.5	66.8			66.8	34.6		70.2	74.7
*Individual characteristics*										
Age, y										
15–24	28.8	35.8	24.7	30.2	32.9	30.5	15.1	16.7	27.4	10.7
25–34	38.1	36.9	42.7	40.8	39.2	41.3	50.8	36.9	49.2	39.8
35+	33.1	27.3	32.6	29.0	27.9	28.2	34.1	46.5	23.4	49.5
Highest schooling level										
None	54.8	32.8	2.5	9.8	0.4	4.5	33.2	1.3	62.7	43.2
Primary	18.9	25.9	48.9	26.2	8.1	55.6	18.1	8.8	18.2	22.3
Secondary or higher	26.3	41.3	48.6	64.1	91.5	40.0	48.7	89.9	19.1	34.5
Parity										
0 children	10.7	22.2	7.3	13.5	28.1	9.6	1.6	18.8	0.1	3.2
1–2 children	28.9	33.5	38.2	32.4	32.0	32.9	14.2	30.0	27.5	50.7
3–4 children	25.9	24.2	33.0	26.8	25.1	26.4	24.5	40.6	36.4	38.3
5 plus children	34.5	20.1	21.5	27.3	14.8	31.0	59.7	10.6	35.9	7.8
*Marital status*										
Married- monogamous	52.3	52.1	72.5	65.3	51.7	59.4	54.5	69.6	63.7	97.2
Married- polygynous	33.7	12.2	10.5	10.3	3.5	22.1	45.2	7.6	35.5	2.7
Not married	13.9	35.8	17.0	24.4	44.8	18.5	0.3	22.9	0.8	0.2
*Contraceptive characteristics*										
Type of method										
Traditional	6.8	21.2	5.5	37.4	45.7	16.7	13.9	33.9	10.8	11.9
Modern	93.2	78.8	94.5	62.6	54.3	83.3	86.1	66.1	89.2	88.1
Method longevity										
Short-acting	52.1	81.3	55.6	75.4	83.1	64.8	64.1	81.1	78.7	38.1
LARC	47.9	18.7	44.4	24.6	16.9	35.2	35.9	18.9	21.3	61.9
Method mix										
Female sterilization	0.4	0.0	4.4	1.8	1.1	3.7	1.5	0.5	1.4	59.1
Male sterilization	0.0	0.0	0.0	0.0	0.0	0.2	0.0	0.0	0.0	0.3
Implants	43.1	16.1	36.2	22.2	15.5	26.9	32.4	13.2	19.2	0.1
IUD	4.4	2.6	3.7	0.6	0.4	4.3	2.0	5.2	0.7	2.4
Injectables	24.5	17.7	33.1	11.9	6.8	30.8	34.4	7.2	31.5	2.0
Pill	8.8	19.4	7.5	6.2	2.8	4.1	13.0	8.5	34.0	6.7
Emergency contraception	0.1	4.8	0.8	3.4	9.6	1.9	0.0	7.1	0.0	0.1
Male condom	11.3	18.1	7.1	14.0	16.2	8.7	1.6	22.8	0.3	17.2
Female condom	0.1	0.0	0.0	0.0	0.1	0.0	0.0	0.0	0.0	0.0
Std. days/cycle beads	0.4	0.0	0.9	1.4	1.7	2.4	1.2	1.1	0.2	0.2
Rhythm	5.8	16.6	3.1	8.1	32.1	5.9	0.3	8.8	0.1	4.6
Withdrawal	0.5	2.3	0.9	22.6	10.4	8.9	8.7	17.6	0.3	5.4
Other traditional	0.5	2.3	1.5	6.8	3.2	2.0	4.9	7.6	10.9	1.9

Type of contraceptive method use differed across sites. Over 90% of users reported using modern methods in Kenya and Burkina Faso, while traditional use was highest in the DRC geographies (37% in Kongo Central and 46% in Kinshasa). Implants and injectables were the most common methods in Burkina Faso, Kenya, and Uganda, while in Côte D'Ivoire the most common methods were injectables and pills. Female sterilization was the most common method reported in Rajasthan. In Kongo Central and Kinshasa, withdrawal and rhythm were the most commonly reported, respectively.

**Fig. 1:**
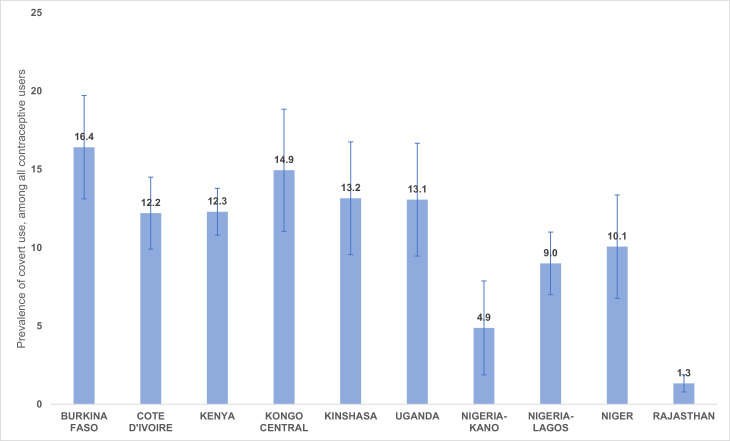
Point estimates and 95% confidence intervals of covert use in 10 Performance Monitoring for Action geographies, 2019/2020.

Prevalence of covert use across the ten geographies ranged from 1% in Rajasthan to 16% in Burkina Faso (Fig. 1). More than one out of every ten users across sites were using covertly in Burkina Faso, Côte D'Ivoire, Kenya, Kongo Central, Kinshasa, Uganda, and Niger. However, there are large 95% confidence intervals for many of the point estimates, which suggests that there may not be true differences in the prevalence of covert use across sites.

### 3.2 Descriptive statistics—Overt vs covert user characteristics

Demographic and contraceptive characteristics differed between overt and covert users across sites (Table 2). In Burkina Faso, Kenya and Kinshasa, larger proportions of women in the lower wealth tertiles were covert users compared to overt users. On the other hand, in Kongo Central, 58% of covert users were in the highest wealth tertile compared to 44% of overt users. Covert users were more likely to be living in rural areas in Burkina Faso compared to overt users, while covert users in Côte D'Ivoire were more likely to be in urban areas than overt users. In Burkina Faso, Côte D'Ivoire, and Kenya covert users had lower educational attainment than overt users. Marital status was significantly associated with type of use across all sites except for Uganda; covert users had higher proportions reporting polygynous marriages compared with overt users in Burkina Faso, Côte D'Ivoire, Kenya, Kongo Central, and Kinshasa. In Côte D'Ivoire, Kenya, Kongo Central, and Kinshasa, covert users were also more likely to report being unmarried than overt users.

**Table 2:** Weighted percentage distributions of characteristics of contraceptive users, by type of use (overt vs covert), in Burkina Faso, Côte D'Ivoire, Kenya, Kongo Central, Kinshasa, and Uganda, 2019/2020

	Burkina Faso			Côte D'Ivoire			Kenya			Kongo Central			Kinshasa			Uganda		
Characteristic	Overt users	Covert users	*p* value	Overt users	Covert users	*p* value	Overt users	Covert users	*p* value	Overt users	Covert users	*p* value	Overt users	Covert users	*p* value	Overt users	Covert users	*p* value
*Household characteristics*																		
Household wealth																		
Lower	27.1	41.8	0.01	20.6	17.9	0.09	30.3	44.0	<0.01	25.9	8.3	0.02	29.9	45.2	<0.01	25.8	28.1	0.57
Middle	30.8	25.9		33.2	46.4		36.9	30.1		30.4	33.7		33.9	31.1		34.3	39.9	
Highest	42.1	32.3		46.1	35.8		32.7	25.9		43.7	57.9		36.2	23.7		39.8	32.0	
Residence																		
Urban	32.4	18.9	0.00	62.0	74.4	0.02	34.0	27.9	0.08	NA	NA	NA	NA	NA	NA	33.9	28.8	0.67
Rural	67.6	81.1		38.0	25.6		66.0	72.1								66.1	71.2	
*Individual characteristics*																		
Age, y																		
15–24	30.9	18.0	0.01	37.0	26.7	0.22	24.5	26.4	0.42	30.5	28.5	0.50	32.5	35.8	0.16	31.5	23.6	0.74
25–34	38.0	38.5		36.1	42.7		43.1	39.2		41.5	36.9		38.2	45.5		40.7	45.3	
35+	31.1	43.4		26.8	30.6		32.4	34.4		28.0	34.6		29.3	18.7		27.8	31.2	
Highest schooling level																		
None	52.6	66.2	0.00	31.2	44.8	0.01	2.3	3.7	<0.01	9.3	12.4	0.39	0.4	0.8	0.25	3.8	8.9	0.44
Primary	18.8	19.7		25.6	27.9		47.7	57.3		27.2	20.5		7.5	11.7		54.9	60.3	
Secondary or higher	28.7	14.1		43.3	27.3		50.0	39.1		63.5	67.1		92.1	87.5		41.3	30.8	
Parity																		
0 children	11.9	4.9	0.00	23.4	13.4	0.14	7.5	5.9	0.01	12.9	17.1	0.73	28.4	25.8	0.19	10.4	4.7	0.62
1–2 children	30.6	20.2		33.1	36.8		38.4	36.9		32.4	32.6		30.8	40.2		33.4	29.7	
3–4 children	25.3	28.9		24.4	22.2		33.7	28.0		26.9	26.1		26.1	18.5		26.5	26.3	
5 plus children	32.2	45.9		19.1	27.7		20.4	29.2		27.9	24.2		14.7	15.5		29.7	39.4	
*Marital status*																		
Married- monogamous	55.3	37.2	0.00	54.7	33.0	0.01	75.0	55.4	<0.01	68.9	44.4	<0.01	55.2	28.0	<0.01	61.7	44.7	0.08
Married- polygamous	30.3	51.3		10.0	27.7		9.6	16.9		8.9	18.5		2.7	8.7		20.5	32.7	
Not married	14.4	11.5		35.3	39.3		15.4	27.7		22.2	37.1		42.0	63.4		17.8	22.7	
*Contraceptive characteristics*																		
Type of method																		
Traditional	6.5	8.6	0.41	22.1	15.1	0.12	5.6	5.0	0.63	39.3	27.0	0.01	47.5	33.6	0.18	17.9	8.9	0.26
Modern	93.5	91.4		77.9	84.9		94.4	95.0		60.7	73.0		52.5	66.4		82.1	91.1	
Method longevity																		
Short-acting	52.9	47.8	0.31	82.0	76.8	0.35	55.1	59.3	0.16	75.2	76.7	0.79	83.4	80.9	0.50	64.7	65.8	0.88
LARC	47.1	52.2		18.0	23.2		44.9	40.7		24.8	23.3		16.6	19.1		35.3	34.2	
Method mix																		
Female sterilization	0.3	1.1	0.00	0.0	0.0	<0.01	4.6	3.1	<0.01	2.1	0.0	<0.01	1.3	0.0	<0.01	4.1	1.1	0.44
Male sterilization	0.0	0.0		0.0	0.0		0.1	0.0		0.0	0.0		0.0	0.0		0.3	0.0	
Implants	42.5	46.6		15.2	22.8		36.6	33.8		22.2	22.5		14.9	19.1		27.3	24.4	
IUD	4.3	4.5		2.9	0.5		3.7	3.8		0.5	0.8		0.4	0.0		3.7	8.7	
Injectables	23.9	27.5		16.6	25.2		31.4	45.2		9.0	28.5		5.3	16.3		28.1	48.4	
Pill	8.5	10.3		18.1	28.8		7.7	6.1		4.6	15.1		1.8	9.4		3.9	6.0	
Emergency contraception	0.1	0.1		4.4	7.7		0.7	1.0		3.1	4.7		8.0	19.9		1.9	1.9	
Male condom	13.5	0.0		20.6	0.0		8.1	0.0		16.5	0.0		18.6	0.0		10.0	0.0	
Female condom	0.2	0.0		0.0	0.0		0.0	0.2		0.0	0.0		0.1	0.0		0.0	0.0	
Std. days/cycle beads	0.2	1.2		0.0	0.0		0.9	0.4		1.5	0.7		1.8	1.3		2.7	0.7	
Rhythm	5.4	8.1		17.5	9.5		3.2	2.8		8.3	6.7		33.9	20.3		6.2	3.5	
Withdrawal	0.6	0.0		2.7	0.0		1.0	0.0		26.6	0.0		12.0	0.0		10.2	0.0	
Other traditional	0.5	0.5		1.9	5.5		1.5	2.2		4.4	20.3		1.7	13.3		1.5	5.4	
																		
*n*	1823	274		943	145		3539	453		619	102		912	148		1078	169	

In all sites except Kongo Central, there were no differences between type of method (traditional vs modern) or method longevity (short acting vs LARC) between covert and overt users. In Kongo Central, covert users had higher proportions of modern use (73%) vs overt users (61%). Method mix was significantly different across all sites between users except in Uganda, although this may largely be driven by the fact that covert users, by definition, were not users of male condoms or withdrawal.

### 3.3 Regressions—covert vs overt user characteristics

The logistic regression results (Table 3) demonstrate that once adjusted, there were only 2 user characteristics that were consistently associated with use type across sites. Women who were in polygynous unions displayed higher odds of using covertly compared to women who were monogamous unions (aORs ranged from 1.8 [95% CI 1.2–2.7] in Burkina Faso to 6.2 in Kinshasa [95% CI 2.9–13.3]). Nonmarried, noncohabiting women with partners were also more likely to be using covertly across all sites (aORs ranged from 2.2 [95% CI 1.0–4.7] in Uganda to 4.4 [95% CI 1.7–11.0] in Kinshasa).

**Table 3:** Adjusted odds ratios for characteristics associated with covert use compared to overt use, in Burkina Faso, Côte D'Ivoire, Kenya, Kongo Central, Kinshasa, and Uganda, 2019/2020

Characteristic		Country, adjusted odds ratio (95% confidence interval)					
		Burkina Faso	Côte D'Ivoire	Kenya	Kongo Central	Kinshasa	**Uganda**
Wealth	Lowest tertile (ref)	–	–	–	–	–	–
	Middle tertile	0.6 (0.4, 1.0)	0.9 (0.5, 1.8)	0.6 (0.4, 0.8)	3.0 (0.9, 10.0)	**0.6 (0.4, 1.0)**	1.1 (0.6, 2.1)
	Highest tertile	0.9 (0.5, 1.7)	0.5 (0.2, 1.0)	0.7 (0.5, 0.9)	**3.8 (1.2, 12.2)**	**0.5 (0.3, 0.8)**	0.9 (0.2, 4.3)
Residence	Rural (ref)	–	–	–	–	–	–
	Urban	0.6 (0.4, 1.1)	**2.6 (1.4, 4.8)**	0.9 (0.7, 1.3)			1.0 (0.4, 2.4)
Age	Age 15–24 (ref)	–	–	–	–	–	–
	Age 25–34	1.1 (0.6, 2.2)	**1.9 (1.0, 3.5)**	1.0 (0.7, 1.5)	1.9 (1.0, 3.8)	1.7 (0.8, 3.4)	1.4 (0.1, 13.9)
	Age 35 plus	1.2 (0.6, 2.5)	1.7 (1.0, 2.9)	1.0 (0.6, 1.6)	**3.3 (1.5, 7.2)**	0.8 (0.3, 2.2)	1.3 (0.2, 10.0)
Education	None (ref)	–	–	–	–	–	–
	Primary	1.2 (0.6, 2.1)	0.9 (0.6, 1.5)	1.0 (0.5, 1.9)	0.8 (0.4, 1.6)	1.0 (0.1, 10.4)	0.4 (0.1, 1.8)
	Secondary plus	0.7 (0.3, 1.4)	**0.5 (0.3, 0.9)**	0.7 (0.3, 1.3)	0.7 (0.3, 1.8)	0.7 (0.1, 6.2)	0.3 (0.0, 3.6)
Parity	0–1 children (ref)	–	–	–	–	–	–
	2–3 children	2.0 (0.9, 4.4)	1.2 (0.6, 2.3)	0.9 (0.6, 1.3)	0.6 (0.2, 1.3)	1.1 (0.5, 2.4)	1.1 (0.2, 6.7)
	4 plus children	2.5 (0.9, 6.8)	0.9 (0.4, 2.0)	1.2 (0.7, 2.1)	0.4 (0.2, 1.1)	2.0 (0.8, 4.9)	1.1 (0.2, 6.8)
Marital status	Married- monogamous (ref)	–	–	–	–	–	–
	Married- polygynous	**1.8 (1.2, 2.7)**	**4.3 (1.7, 10.7)**	**2.2 (1.5, 3.2)**	**3.0 (1.3, 7.2)**	**6.2 (2.9, 13.3)**	**2.1 (1.1, 3.9)**
	Not married	**3.0 (1.5, 6.2)**	**3.0 (1.5, 5.8)**	**3.0 (2.0, 4.5)**	**2.4 (1.2, 4.8)**	**4.4 (1.7, 11.0)**	**2.2 (1.0, 4.7)**
							
*N*		2093	1076	3950	714	1039	1175

Notes: Boldfaced estimates indicate *p* < 0.05. The PMA sample in Kinshasa is urban only. The sample in Kongo Central includes both urban and rural areas, but urban/rural residence is not included in the data for Kongo Central as it is missing from the sampling frame.

Wealth was associated with covert use in Kenya, Kongo Central, and Kinshasa in the adjusted models. Women in the wealthiest tertile had lower odds of using covertly compared to women in the lowest tertile in Kenya and Kinshasa (aORs 0.7 [95% CI 0.5–0.9] and 0.5 [95% CI 0.3–0.8], respectively). In contrast, in Kongo Central, wealthier women were more likely to use covertly (aOR 3.8 [95% CI 1.2–12.2]). Women in urban settings were more likely to use covertly compared to those in rural settings in Côte D'Ivoire (aOR 2.6 [95% CI 1.4–4.8].

Age was related to type of use in 2 geographies; in Côte D'Ivoire and Kongo Central, older women were more likely to use covertly than younger women (age 25+ in Côte D'Ivoire, age 35+ in Kongo Central vs age 15–24 years). Higher education only remained associated with reduced odds of covert use in Côte D'Ivoire in the fully adjusted models, where having a secondary or higher education was associated with decreased odds of using covertly, compared to no education (aOR 0.5 [95% CI 0.3−0.9]).

Finally, our sensitivity analysis among married women that included a measure on relationship length showed similar results to the main models (Appendix Table A 1). While some of results seen in the main model were attenuated, polygamous marriage was still consistently associated with greater odds of using covertly than monogamous marriage. Across sites there was a negative relationship with relationship age and covert use; however, this association only achieved statistical significance in Kenya, where women who had been married 10+ years were less likely to be using covertly than those who had been married 0 to 4 years (aOR 0.6 [95% CI 0.4–1.0]). Relationship length may not be indicative of relationship quality, for which better measures are needed.

**Table A 1:** Adjusted odds ratios for characteristics associated with covert use compared to overt use, in Burkina Faso, Côte D'Ivoire, Kenya, Kongo Central, Kinshasa, and Uganda, 2019/2020

Characteristic		Country, adjusted odds ratio (95% confidence interval)					
		Burkina Faso	Côte D'Ivoire	Kenya	Kongo Central	Kinshasa	**Uganda**
Wealth	Lowest tertile (ref)	–	–	–	–	–	–
	Middle tertile	**0.6 (0.3, 0.9)**	1.5 (0.7, 3.0)	**0.5 (0.3, 0.7)**	3.3 (0.9, 12.1)	0.7 (0.3, 1.6)	1.0 (0.5, 2.1)
	Highest tertile	1.0 (0.5, 1.9)	0.5 (0.2, 1.3)	**0.6 (0.4, 0.9)**	**4.6 (1.2, 17.7)**	0.9 (0.3, 2.2)	0.8 (0.2, 4.4)
Residence	Rural (ref)	–	–	–	–	–	–
	Urban	0.7 (0.4, 1.2)	**2.6 (1.4, 4.8)**	1.1 (0.7, 1.5)			0.9 (0.3, 2.1)
Age	Age 15–24 (ref)	–	–	–	–	–	–
	Age 25–34	1.1 (0.5, 2.6)	1.5 (0.6, 3.5)	1.1 (0.8, 1.7)	1.9 (0.7, 4.8)	2.5 (0.7, 8.9)	1.1 (0.3, 4.0)
	Age 35 plus	1.0 (0.4, 2.7)	1.8 (0.8, 4.4)	1.4 (0.8, 2.5)	**4.5 (1.4, 14.0)**	1.3 (0.3, 6.1)	1.3 (0.3, 4.7)
Education	None (ref)	–	–	–	–	–	–
	Primary	1.1 (0.6, 2.1)	1.1 (0.5, 2.2)	1.0 (0.5, 2.0)	0.6 (0.3, 1.5)	0.1 (0.0, 2.2)	0.4 (0.1, 1.7)
	Secondary plus	0.8 (0.4, 1.9)	0.9 (0.4, 2.1)	0.7 (0.3, 1.6)	0.6 (0.2, 1.4)	0.0 (0.0, 1.1)	0.4 (0.0, 4.5)
Parity	0–1 children (ref)	–	–	–	–	–	–
	2–3 children	2.0 (0.8, 4.5)	0.6 (0.3, 1.5)	0.7 (0.4, 1.2)	0.5 (0.2, 1.6)	0.6 (0.2, 2.1)	0.8 (0.2, 2.5)
	4 plus children	2.7 (1.0, 7.9)	0.8 (0.2, 2.6)	1.3 (0.7, 2.4)	**0.3 (0.1, 1.0)**	1.4 (0.3, 6.2)	1.2 (0.3, 4.4)
Marital status	Married- monogamous (ref)	–	–	–	–	–	–
	Married- polygynous	**1.9 (1.3, 3.0)**	**4.4 (2.0, 9.4)**	**2.1 (1.4, 3.0)**	**3.2 (1.3, 7.8)**	**8.8 (3.7, 21.0)**	**2.2 (1.2, 4.1)**
Length of marriage	0–4 y (ref)	–	–	–	–	–	–
	5–9 y	0.9 (0.4, 2.0)	0.5 (0.2, 1.4)	0.9 (0.6, 1.5)	0.7 (0.2, 2.4)	0.9 (0.2, 3.7)	0.8 (0.1, 5.1)
	10+ y	0.9 (0.4, 2.2)	0.4 (0.1, 1.3)	**0.6 (0.4, 1.0)**	0.8 (0.2, 3.0)	0.5 (0.1, 2.4)	0.6 (0.1, 2.5)
							
*N*		1574	674	3222	513	585	947

Notes: Boldfaced estimates indicate *p* < 0.05. The PMA sample in Kinshasa is urban only. The sample in Kongo Central includes both urban and rural areas, but urban/rural residence is not included in the data for Kongo Central as it is missing from the sampling frame.

## 4 Discussion

This study generates updated, representative estimates of covert use across multiple geographies using the direct estimation technique. Our results show that covert users represent a small but important proportion of users across all sites, ranging from 1% of users in Rajasthan to 16% in Burkina Faso. While scholars have hypothesized that covert use would decrease as overall contraceptive levels increase [2], our findings do not unequivocally support that proposition, reinforcing the fact that these women represent an important subgroup of users, despite increasing contraceptive prevalence in many of these sites.

Relationship status was the only characteristic consistently associated with using covertly across settings. Our finding that married women in polygynous unions were more likely to be using covertly than their monogamous counterparts has been seen in prior studies [5]. We also found that single women in relationships were more likely to be using covertly compared to married women in monogamous unions, echoing previous work that has included nonmarried women in their samples [7,9,16,17]. The exact factors driving the association between relationship status and covert use may differ between subpopulations. Women in polygynous unions may use covertly if they feel unsupported financially and burdened by childcare [5,12]. Conversely, women in more casual relationships may believe family planning to be in the realm of female decision-making rather than one to be made jointly [3]. Or it may be that women in both polygynous and casual relationships have less bargaining power within the relationship compared to women in monogamous relationships, and are therefore less able to communicate and collaborate around decisions on contraceptive use [18].

Prior studies have shown that education and wealth [4,7,9] were protective against covert use compared to overt use, relationships that were present in Côte D'Ivoire, Kenya, and Kinshasa. While across all sites, women with secondary or higher education were less likely to be using covertly than overtly, this relationship only achieved statistical significance in Côte D'Ivoire. That these 2 proxies for empowerment are associated with lower likelihood of covert use may support the hypothesis that covert users are less empowered than overt users. However, that these patterns are not seen uniformly across sites implies that these relationships are complex and context specific; socio-demographics alone may not explain whether covert use is an action taken in response to disempowerment or if it is an expression of empowerment.

This study has several limitations. First, there are limitations in our measurement of covert use. The direct question on partner knowledge is thought to be an underestimate of covert use[1], therefore our study may be missing women who were not comfortable sharing their use with survey interviewers, potentially excluding the most vulnerable women. It is unknown in which direction these biases may influence our findings. Additionally, PMA surveys do not interview male partners, and it is possible men's men’ knowledge differs from what their partners report. Finally, our study is missing important characteristics of relationship dynamics that capture power differentials, especially in the context of unmarried partnerships, which could be particularly important when thinking about potential transactional relationships.

Our findings can inform family planning research, practice, and interventions. We find that relationship status is a consistent predictor of covert use in multiple contexts, but we also find that the pattern is consistent with both more and less power over contraceptive choices within relationships. Covert use could be simultaneously driven both by relationship status of comparative economic and social vulnerability, or social and relational empowerment. Most likely, both drivers are at play. There is a need for more research on covert use and relationship quality, including a concerted need for reliable measures surrounding relationship quality, to help inform when and how women's contraceptive choices are expanded or constrained by the dynamics of their relationship.

Moreover, familiarity with the profiles of covert users can assist health care providers to counsel women on the most appropriate methods, manage side effects, and maintain discretion through strategies like integration into maternal and child health care services [5,16]. Community-based strategies, such as the employment of health surveillance assistants or community health volunteers, could further help women obtain and continue contraceptive use, while maximizing confidentiality as they would not need to seek formal services [19].

Given the small, but important prevalence of covert use, interventions that involve partners and men are needed to alter the underlying environments that necessitate covert use in the first place. Improving spousal communication around family planning may reduce the need for women to use covertly and support those who desire overt use; partner support and discussions have been shown to be predictive of both adoption and continuation [20] . Interventions that increase knowledge about family planning and the benefits of birth spacing, particularly for male partners, may help to increase acceptability, reduce stigma and increase overt use [13,21].

Despite efforts to involve male partners in the hopes of reducing covert use, protecting women who choose to use contraception without their partner's knowledge is essential. Given that these women are regaining some reproductive autonomy in contexts where it may be explicitly threatened, supporting, and facilitating their access to family planning is crucial, while concurrently working towards larger social norms changes in the longer term.

## Footnotes

1 The PMA sample in Kinshasa is urban only. The sample in Kongo Central includes both urban and rural areas, but urban/rural residence is not included in the data for Kongo Central as it is missing from the sampling frame.
